# Botulinum Toxin Type A Inhibits Submandibular Secretion via the ERK/miR-124-3p/Specificity Protein 1/Claudin-1 Axis

**DOI:** 10.3390/cells14171366

**Published:** 2025-09-02

**Authors:** Qian-Ying Mao, Yan Huang, Zhuo Chen, Xiao-Feng Shan, Shang Xie, Li-Ling Wu, Ruo-Lan Xiang, Zhi-Gang Cai

**Affiliations:** 1 National Center for Stomatology, National Clinical Research Center for Oral Diseases, National Engineering Research Center of Oral Biomaterials and Digital Medical Devices, Peking University School and Hospital of Stomatology, No. 22 Zhong Guan Cun South Street, Haidian District, Beijing 100081, China; maoqianying0418@126.com (Q.-Y.M.); kgsxf@263.net (X.-F.S.); xs2013@hsc.pku.edu.cn (S.X.); 2 Xiamen Key Laboratory of Stomotalogical Disease Diagnosis and Treatment, Department of Oral and Maxillofacial Surgery, Stomatological Hospital of Xiamen Medical College, Xiamen 361102, China; hynh@bjmu.edu.cn; 3 Key Laboratory of Molecular Cardiovascular Sciences, Department of Physiology and Pathophysiology, Ministry of Education, Peking University School of Basic Medical Sciences, No. 38 Xueyuan Road, Haidian District, Beijing 100191, China; 2111210002@bjmu.edu.cn (Z.C.); pathophy@bjmu.edu.cn (L.-L.W.)

**Keywords:** botulinum toxin type A, miR-124-3p, specific protein 1, tight junction, submandibular gland

## Abstract

Botulinum toxin type A (BTXA) is widely used for the treatment of sialorrhea; however, its mechanism remains unclear. Tight junctions (TJs) are limiting factors for salivary secretion through the paracellular pathway in the salivary gland, among which claudin-1 (Cldn1) is a TJ protein that mainly plays a barrier role. This study observed that Cldn1 was upregulated in BTXA-treated rats’ submandibular glands and SMG-C6 cells. Knockdown of Cldn1 reversed the BTXA-induced reduction in paracellular permeability. The transcription factor specificity protein-1 (Sp1), which binds to the Cldn1 promoter, was also upregulated by BTXA, and its expression was linked to the ERK1/2 pathway. Inhibition of ERK1/2 by U0126 reversed the BTXA-induced upregulation of Sp1 and Cldn1, as well as the reduction in paracellular permeability. MiR-124-3p, which directly targets Sp1, was downregulated by BTXA, but its overexpression counteracted Sp1 and Cldn1 upregulation. Although miR-124-3p did not affect ERK1/2 phosphorylation, ERK1/2 inhibition reversed the BTXA-induced decrease in miR-124-3p expression. These findings reveal a regulatory pathway through which BTXA reduces paracellular permeability in SMG-C6 cells via the ERK1/2/miR-124-3p/Sp1/Cldn1 axis.

## 1. Introduction

Sialorrhea, characterized by excessive saliva spilling beyond the lip’s edge, typically results from hypersalivation or swallowing disorders. Sialorrhea commonly occurs in numerous neurological diseases, including cerebral palsy, Parkinson’s disease, and amyotrophic lateral sclerosis [1,2]. Prolonged sialorrhea can cause skin irritation, infections, aspiration pneumonia, and even psychosocial disorders, affecting social interactions and mental health [3]. Botulinum toxin type A (BTXA) is a neurotoxin with a molecular weight of 150,000 kDa that is produced by the Gram-positive anaerobic bacterium Clostridium botulinum. Since the 1990s, various clinical studies have confirmed the efficacy of injecting BTXA into the salivary glands to treat sialorrhea, a process that involves the cleavage of synaptosomal-associated protein 25 (SNAP-25) to inhibit acetylcholine release. Recent studies suggest that BTXA directly affects acinar cells by inhibiting autophagic flow [4] and altering the distribution of aquaporin 5 [5]. However, the nascent stage of these foundational studies restricts broader clinical applications. Therefore, in-depth elucidation of the mechanism underlying the BTXA-induced inhibition of salivary secretion is necessary.

Tight junctions (TJs) are epithelial intercellular junctions that mediate cell-to-cell interactions and act as selective permeability barriers. Tight junctions in the salivary gland epithelium serve as the structural basis for salivary secretion via the paracellular pathway [6]. Tight junctions are macromolecular protein complexes consisting of claudins (Cldns), occludin, and members of the zonula occludens (ZO) family [7]. Claudins, which interact with the ZO family and the cytoskeletal component F-actin, are pivotal in paracellular transport. The 27 isoforms of Cldns are categorized into barrier-forming and pore-forming types [8]. Most studies indicate that claudin-1 (Cldn1) functions primarily as a barrier. Claudin-1 is essential for maintaining the barrier function of the skin in the epidermis. Claudin-1 knockout mice lose their tight junction barrier to water and macromolecules and consequently die of dehydration during the neonatal period [9]. Knockdown of Cldn1 in human keratinocytes compromises the seal against molecules, such as fluorescein and larger molecules like 4 kDa and 40 kDa FITC–dextran [10]. Knockdown of Cldn1 in hepatocellular carcinoma cells and normal rat cholangiocyte cells increases paracellular permeability [11]. However, the role of Cldns in the BTXA-induced inhibition of salivary secretion in submandibular glands remains unclear.

MicroRNAs (miRNAs), small noncoding RNAs of 19–24 nucleotides, play a critical role in gene regulation by inhibiting translation or facilitating mRNA degradation, primarily by binding to the 3′-untranslated region (3′-UTR) of their target mRNAs [12]. Previous studies have demonstrated that miRNAs play important roles in the regulation of TJ protein expression as well as epithelial and endothelial barrier functions. In a mouse model of hypoxia-induced brain injury, miR-212 and miR-132 impair blood-brain barrier function by targeting Cldn1, ligand adhesion molecule 3 and TJ-associated protein 1 [13]. MiR-3571 regulates vascular smooth muscle cell proliferation and migration by targeting Cldn1 [14]. In patients with irritable bowel syndrome, miR-29 has been shown to increase intestinal permeability by regulating Cldn1 expression [15]. Furthermore, studies have shown that BTXA can influence the expression of miRNAs. For example, miR-133a/b and miR-1/206 are linked to BTXA-induced skeletal muscle paralysis [16]. Additionally, BTXA induces epithelial-mesenchymal transition and autophagy in keloid fibroblasts by regulating miRNAs such as miR-1587 and miR-2392, which target the zinc finger E-box-binding homeobox [17]. However, further investigations are needed to determine whether BTXA can inhibit salivary secretion by affecting miRNA expression.

This study aimed to explore alterations in Cldns and the potential mechanisms by which miRNAs influence BTXA-inhibited salivary secretion. Our previous miRNA-seq analyses revealed that BTXA reduced miR-124-3p expression in submandibular glands (SMGs), suggesting its involvement in BTXA-induced inhibition of salivary secretion [18]. Bioinformatic predictions further indicated that miR-124-3p can directly bind to the 3′-UTR of Sp1. As a transcription factor, Sp1 has been reported to bind to the Cldn1 promoter and promote its expression [19]. Moreover, the MAPK pathway, specifically the ERK1/2, has been shown to regulate miRNA biogenesis. Therefore, we focused on the ERK/miR-124-3p/Sp1/Cldn1 axis to investigate the signaling pathway through which BTXA inhibits salivary secretion.

## 2. Materials and Methods

### 2.1. Animals

All animal experimental procedures were approved by the Ethics Committee of Animal Research, Peking University Health Science Center (No. LA2020067) and followed ARRIVE guidelines. Male Sprague-Dawley rats (230–250 g) were acquired from the Laboratory Animal Service Center, Peking University Health Science Center. After anesthesia with 5% isoflurane in oxygen (1 L/min) and maintenance at 2–2.5% isoflurane in oxygen (0.2 L/min), the left submandibular glands (SMGs) were exposed via a median cervical incision. The BTXA group received 6 U BTXA (Lanzhou Biological Co., Lanzhou, China) in 0.1 mL normal saline per SMG, and the control group received 0.1 mL saline. Extracted SMGs were immediately frozen in liquid nitrogen and stored at −80 °C.

### 2.2. Measurement of Saliva Secretion

Rats were fasted for at least 5 h with free access to water. After anesthesia, the SMG duct was isolated and placed in a capillary tube (inner diameter 1 mm; length 100 mm) under microscopic observation. Saliva was collected for 10 min starting 5 min after pilocarpine injection (10 μg/g body weight). The saliva volume was calculated from the liquid level in the capillary tube.

### 2.3. Cell Culture

The rat SMG cell line SMG-C6 (a gift from Dr. David O. Quissell) was cultured in DMEM/F12 (1:1) supplemented with 2.5% fetal bovine serum; 5 μg/mL, transferrin; 1.1 μmol/L, hydrocortisone; 0.1 μmol/L, retinoic acid; 2 nmol/L, thyronine T3; 5 μg/mL, insulin; 80 ng/mL, epidermal growth factor; 50 μg/mL, gentamicin sulfate; 5 mol/L, glutamine; 100 U/mL, penicillin; and 100 mg/L, streptomycin. All reagents were from Thermo Fisher Scientific (Waltham, MA, USA) and Sigma-Aldrich (St. Louis, MO, USA). BTXA was prepared at 50 U/mL in culture medium.

### 2.4. Transmission Electron Microscopy

The SMGs were fixed in 2.5% glutaraldehyde at 4 °C overnight, then post-fixed in 1% buffered osmium tetroxide, sectioned with ultramicrotome and stained with 10% uranyl acetate and 1% lead citrate. The sections were examined using transmission electron microscope (HITACHI H-7000, Tokyo, Japan). For the morphometric analysis, the width of the apical TJs was assessed by measuring the distance between adjacent TJs. For morphometry, the width of apical TJs was measured between adjacent TJs in three sections from five random fields per section for both groups. Measurements were averaged using ImageJ 1.54g by two blinded examiners.

### 2.5. Histological and Immunofluorescence Staining

Submandibular gland samples were fixed in 4% paraformaldehyde, embedded in paraffin, and sections were H&E-stained. Histological analyses were conducted using a microscope (EVOS FL AUTO, life technologies, Carlsbad, CA, USA).

For immunofluorescence staining, the slices (7 µm) were blocked with bovine serum albumin and incubated with primary antibodies against Cldn1 (1:100, Bioworld Technology, St. Louis Park, MN, USA), Cldn3 (1:100, Bioworld Technology, St. Louis Park, MN, USA), Cldn4 (1:100, Bioworld Technology, St. Louis Park, MN, USA), Cldn7 (1:100, Bioworld Technology, St. Louis Park, MN, USA), Cldn10 (1:100, Thermo Fisher Scientific, Waltham, MA, USA), and Sp1 (1:100, proteintech, Wuhan, China). Images were captured with a laser scanning confocal microscope (Leica TCS SP8, Wetzlar, Germany).

### 2.6. Measurement of Transepithelial Electrical Resistance and Paracellular Tracer Flux

SMG-C6 cells were seeded at a density of 1 × 10^5^ cells/cm^2^ on Corning Transwell™ Filters (6.5 mm diameter, 0.4 μm pore size) (Corning, New York, NY, USA). Transepithelial electrical resistance (TER) determination was performed using an EVOM2 (World Precision Instruments, Sarasota, FL, USA) after 4–5 days, once the cells grew into a confluent monolayer. Final results were calculated by subtracting the blank filter value (90 Ω) from the TER value and then multiplying by the filter’s surface area. For the paracellular tracer flux assay, 4 kDa or 40 kDa FITC-dextran (1 g/L) was added to the P buffer (containing 10 mM HEPES/NaOH, pH 7.4, 1 mM sodium pyruvate, 10 mM glucose, 3 mM CaCl_2_, and 145 mM NaCl) in the basal compartment. After 3 h of incubation, samples were collected from the apical side, and the fluorescence intensity was measured using EnSpire Multilabel Plate Reader (PerkinElmer, Waltham, MA, USA).

### 2.7. Knockdown of Cldn1 and Sp1

The Cldn1 and Sp1 specific siRNAs and negative controls were obtained from HanBio company (Wuhan, China). The sequences are shown in Table 1. For transfection, SMG-C6 cells were cultured to 60–70% confluency. Transfection of siRNAs (50 nmol/L) was performed using Lipofectamine^®^ 2000 (Thermo Fisher Scientific, Waltham, MA, USA) following the manufacturer’s protocol.

### 2.8. Transfection of miR-124-3p Mimic and Inhibitor

SMG-C6 cells (60–70% confluent) were transfected with miR-124-3p mimic (50 nmol/L), inhibitor (100 nmol/L), or controls using ribo FECT™ CP Kit (RiboBio, Guangzhou, China).

### 2.9. Plasmid Construction and Dual-Luciferase Activity Assay

According to the previous study [14], Cldn1 promoter sequence (−284 to −84 bp) with wild-type or mutant binding sites was cloned into pGL3 basic vector. Sp1 3′UTR sequences with wild-type or mutant miR-124-3p binding sites were inserted into pmiR-Report vector. After 48 h transfection with Lipofectamine^®^ 2000, luciferase activity was measured with the dual-luciferase reporter assay system (Promega, Madison, WI, USA) and detected with EnSpire Multilabel Plate Reader (PerkinElmer, Waltham, MA, USA).

### 2.10. Western Blot Analysis

Total proteins were extracted using RIPA buffer (Thermo Fisher Scientific, Waltham, MA, USA). Equal amounts of protein (20 μg) were separated on 10% or 12% SDS-PAGE, transferred to PVDF membranes, blocked with 5% milk, and probed with primary antibodies overnight at 4 °C, followed by incubation with peroxidase-conjugated secondary antibodies. Bands were visualized with ECL reagent (Thermo Fisher Scientific, Waltham, MA, USA) and quantified using Image J v1.8.0.

The primary antibodies used in this study: Cldn1, Cldn3, Cldn4, Cldn7 (1:1000, Bioworld, St. Louis Park, MN, USA), Cldn10 (1:1000, Thermo Fisher Scientific, Waltham, MA, USA), Sp1 (1:2000, proteintech, Wuhan, China), p-ERK1/2, ERK1/2, p-p38, p38, p-JNK, JNK, and β-actin (1:1000, Cell Signaling Technology, Danvers, MA, USA).

### 2.11. RNA Extraction and Quantitative Real-Time PCR

Total RNA was extracted via TRIzol reagent (Sigma-Aldrich, St. Louis, MO, USA). Reverse transcription for miRNA analysis was performed using 1 µg of total RNA with the miRNA 1st Strand cDNA Synthesis Kit (Vazyme, Nanjing, China). Quantitative real-time RT-PCR (qRT-PCR) amplification was performed by miRNA Universal SYBR qPCR Master Mix (Vazyme, Nanjing, China). For mRNA detection, reverse transcription of 1 μg total RNA using HiScript II Q RT SuperMix for qPCR (Vazyme, Nanjing, China). The cDNAs were amplified by qRT-PCR using SYBR Green Master Mix (Vazyme, Nanjing, China). The U6 and β-actin were used as internal control for miRNA and mRNA detection, respectively. All the primers used are listed in Table 2.

### 2.12. Statistical Analysis

Data are shown as mean ± standard error of mean (SEM). For comparisons between two groups, statistical analysis was performed using Student’s *t*-test. Comparisons among multiple groups were conducted using one-way or two-way analysis of variance (ANOVA), followed by Tukey’s test. All analyses were carried out with GraphPad Prism 8.0, and statistical significance was defined as *p* < 0.05.

## 3. Results

### 3.1. Upregulation of Cldn1 and Cldn3 Expression Following BTXA-Induced Inhibition of Salivary Secretion in the Submandibular Gland

**Figure 1:**
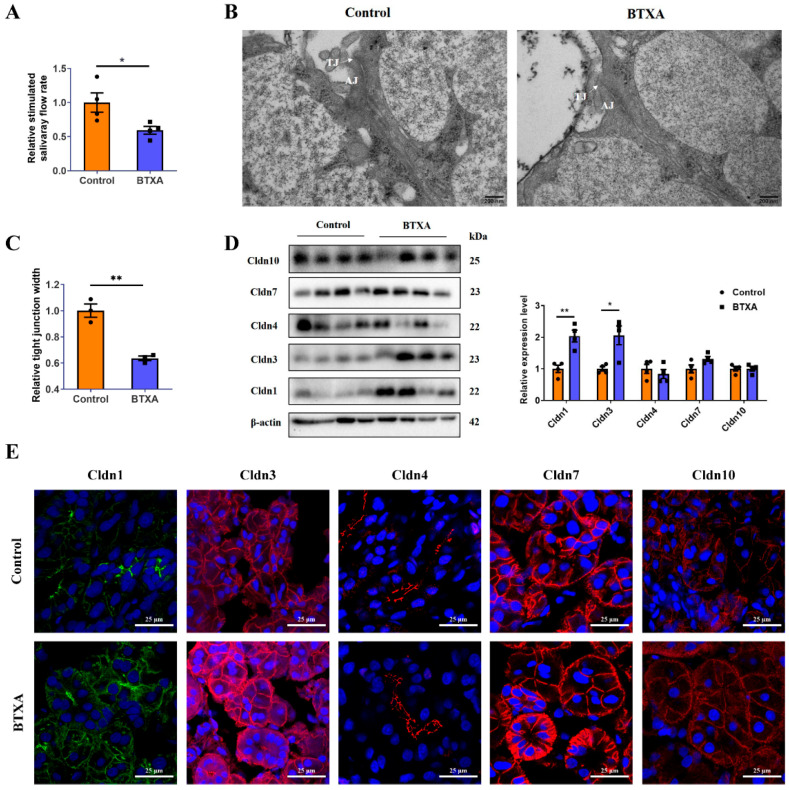
Claudin-1 and Cldn3 expression is upregulated in the BTXA group. (**A**) Stimulated salivary flow rates of control and BTXA group. (**B**) Transmission electron microscopy images of TJs (white arrows) in the SMGs of control and BTXA group. (**C**) Statistical analysis of TJ width in the rat SMGs. (**D**) The protein levels of Cldn1, Cldn3, Cldn4, Cldn7, and Cldn10 between the control and BTXA group. (**E**) Immunofluorescence staining of Cldn1, Cldn3, Cldn4, Cldn7, and Cldn10 in SMGs of the control and BTXA group. TJ, tight junction; AJ, adherens junction; SMG, submandibular gland; Cldn, claudin. Data are presented as mean ± SEM; *n* = 3–4, * *p* < 0.05; ** *p* < 0.01 compared with control group.

Following BTXA injection, the salivary flow rate in the SMG decreased, as demonstrated in Figure 1A, indicating the inhibitory effect of BTXA on salivary secretion. Transmission electron microscopy images revealed that in the control group, the TJs between adjacent acini were located in the apical region, forming a narrow void, whereas the basal region comprised adhesive junctions. Conversely, in the BTXA-treated group, the TJ structure appeared blurred and exhibited a reduced electron density (Figure 1B). Quantitative analysis revealed that the average width of TJs, an indicator of TJ opening, was decreased in the BTXA group (Figure 1C). The expression and distribution of the Cldn family, which is essential for TJ structure and function, were analyzed. Notably, the protein levels of Cldn1 and Cldn3 increased after BTXA treatment, whereas the levels of Cldn4, Cldn7, and Cldn10 remained relatively unchanged (Figure 1D). Immunofluorescence staining showed that Cldn1 and Cldn3 were expressed mainly on the apicolateral and basolateral membranes of the acini and ducts in the rat SMG, and their staining intensities were increased in the BTXA group. Cldn4 was located mainly on the apicolateral membranes of ducts. Cldn7 was present on the apicolateral and basolateral membranes of acini and ducts. Cldn10 was expressed on the apicolateral and basolateral membranes of acini. However, no obvious differences were observed in the distribution of Cldn4, Cldn7, and Cldn10 (Figure 1E).

### 3.2. BTXA Increases the Expression of Cldn1 and Cldn3 and Decreases Paracelluar Permeability in SMG-C6 Cells

**Figure 2:**
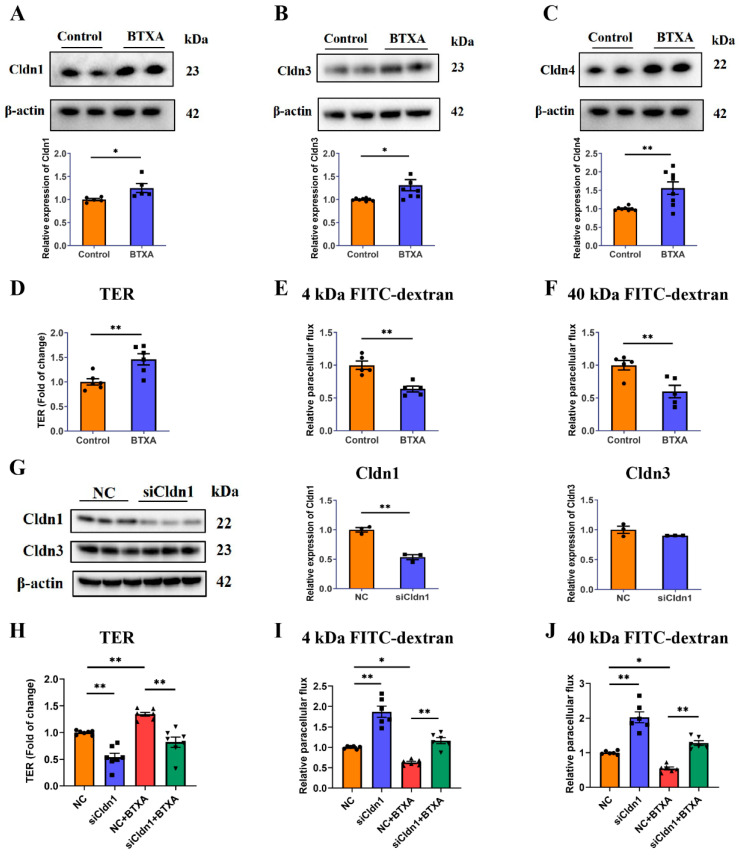
Claudin-1 is required for BTXA-induced reduction in paracellular permeability. (**A**–**C**) The protein levels of Cldn1 (**A**), Cldn3 (**B**), and Cldn4 (**C**) in BTXA-treated SMG-C6 cells. (**D**) The effect of BTXA on the TER values of SMG-C6 cells. (**E**,**F**) The effect of BTXA on the paracellular flux of 4 kDa (**E**) and 40 kDa FITC-dextran (**F**). (**G**) The protein levels of Cldn1 and Cldn3 after transfecting SMG-C6 cells with Cldn1-specific siRNA. (**H**) The TER values of Cldn1-knockdown SMG-C6 cells. (**I**,**J**) The paracellular flux of 4 kDa (**I**) and 40 kDa FITC-dextran (**J**) of Cldn1-knockdown SMG-C6 cells. SMG-C6 cells treated with 50 U/mL BTXA for 24 h. TER, transepithelial electrical resistance; Cldn, claudin. Data are presented as mean ± SEM; *n* = 5–8, * *p* < 0.05; ** *p* < 0.01 compared with control group.

We investigated the direct effect of BTXA on the expression of the Cldn family in SMG-C6 cells. Our results revealed increased protein levels of Cldn1 (Figure 2A), Cldn3 (Figure 2B) and Cldn4 (Figure 2C). To determine the effects of BTXA on TJ barrier function, we measured TER values and FITC-dextran permeability. The TER reflects the integrity of the single layer barrier of epithelial cells, with higher values correlating to reduced paracellular pathway permeability. The TER value of SMG-C6 cells increased significantly after BTXA treatment for 24 h (Figure 2D). Dextran, a specific paracellular tracer, can also be used to evaluate paracellular permeability. BTXA treatment resulted in a decrease in the paracellular flux of both 4 kDa (Figure 2E) and 40 kDa FITC-dextran (Figure 2F). These findings suggest that BTXA exerts a direct effect on acinar cells, leading to a decrease in paracellular permeability.

Claudin-1 is considered a key component of the paracellular barrier. Our findings demonstrated that BTXA upregulated Cldn1 expression in both animal and cell experiments. To elucidate the relationship between BTXA-induced Cldn1 overexpression and decreased paracellular permeability, we employed siRNA-mediated Cldn1 knockdown. The Western blot results showed significant reductions in Cldn1 expression in the knockdown group, whereas Cldn3 levels remained unchanged (Figure 2G). Following Cldn1 knockdown, there was a notable decrease in TER values (Figure 2H), along with an increase in the permeability of 4 kDa and 40 kDa FITC-dextran (Figure 2I,J). These results suggest that Cldn1 functions as a barrier in SMG-C6 cells. Additionally, Cldn1 knockdown reversed the BTXA-induced increase in TER values (Figure 2H) and the decrease in the permeability of 4 kDa and 40 kDa FITC-dextran (Figure 2I,J). These findings suggest that BTXA modulate the permeability of the paracellular pathway in SMG-C6 cells by regulating Cldn1.

### 3.3. ERK1/2 Activation Is Involved in BTXA-Induced Reduction in Paracellular Permeability and Upregulation of Cldn1 Expression

**Figure 3:**
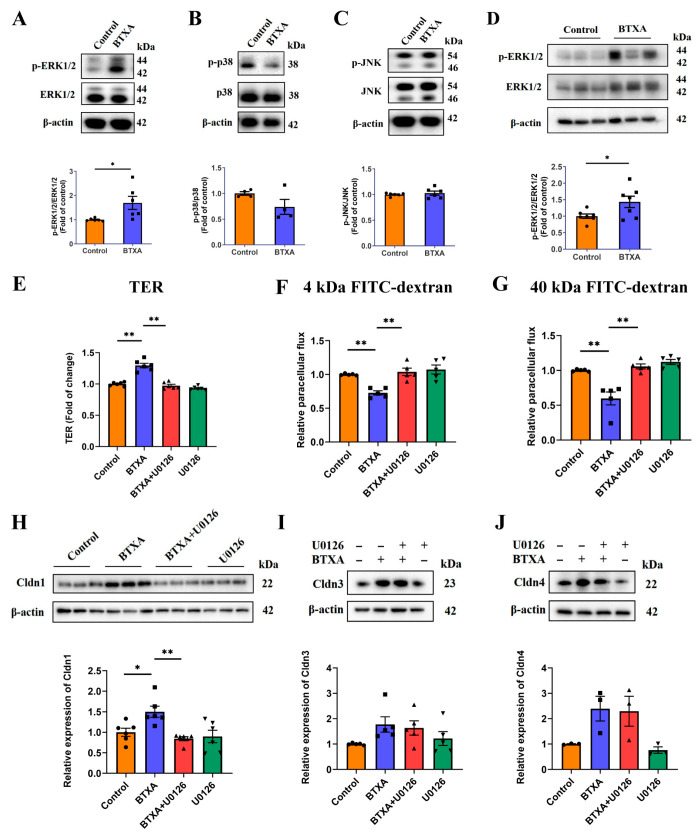
ERK1/2 pathway is activated and involved in BTXA-induced upregulation of claudin-1. (**A**–**C**) Western blot analysis of ERK1/2 (**A**), p38 (**B**), and JNK (**C**) phosphorylation in BTXA-treated SMG-C6 cells. (**D**) Western blot analysis of ERK1/2 phosphorylation in rat SMGs. (**E**) BTXA-induced increase in TER was reversed by preincubation with U0126. (**F**,**G**) BTXA-induced decrease in paracellular flux of 4 kDa (**F**) and 40 kDa FITC-dextran (**G**) was reversed by preincubation with U0126. (**H**) BTXA-induced upregulation of Cldn1 was reversed by preincubation with U0126, as detected by Western blot. (**I**,**J**) BTXA-induced upregulation of Cldn3 and Cldn4 remained unchanged after preincubation with U0126, as detected by Western blot. SMG-C6 cells treated with 50 U/mL BTXA for 24 h. TER, transepithelial electrical resistance; Cldn, claudin. Data are presented as mean ± SEM; *n* = 3–7, * *p* < 0.05; ** *p* < 0.01 compared with control group.

The mitogen-activated protein kinase (MAPK) signaling pathway is crucial for regulating the expression of genes encoding TJ associated proteins. First, we investigated the effects of BTXA on MAPK signaling pathways in SMG-C6 cells. Our results indicated that BTXA treatment specifically activated the ERK1/2 pathway (Figure 3A), with no notable activation observed in the p38 and JNK pathways (Figure 3B,C). Additionally, an increase in ERK1/2 phosphorylation was observed in the SMGs following BTXA treatment (Figure 3D).

To elucidate the role of ERK1/2 activation in the BTXA-mediated inhibition of paracellular permeability, we employed U0126, an inhibitor of the ERK1/2 pathway. The results showed that inhibiting the ERK1/2 pathway reversed the increase in TER values (Figure 3E) and the BTXA-induced decrease in the permeability of 4 kDa and 40 kDa FITC-dextran (Figure 3F,G). These findings suggest that ERK1/2 activation is involved in the reduction in paracellular permeability of BTXA.

Further, we examined the effects of BTXA-induced ERK1/2 activation on TJ molecules. Western blot analysis revealed that U0126 reversed the BTXA-induced upregulation of Cldn1 (Figure 3H), while the expression levels of Cldn3 and Cldn4 remained unaffected (Figure 3I,J). These findings suggest that the ERK1/2 pathway mediates upregulation of Cldn1 expression by BTXA.

### 3.4. BTXA Increases the Expression of Sp1 in Submandibular Gland and SMG-C6 Cells

**Figure 4:**
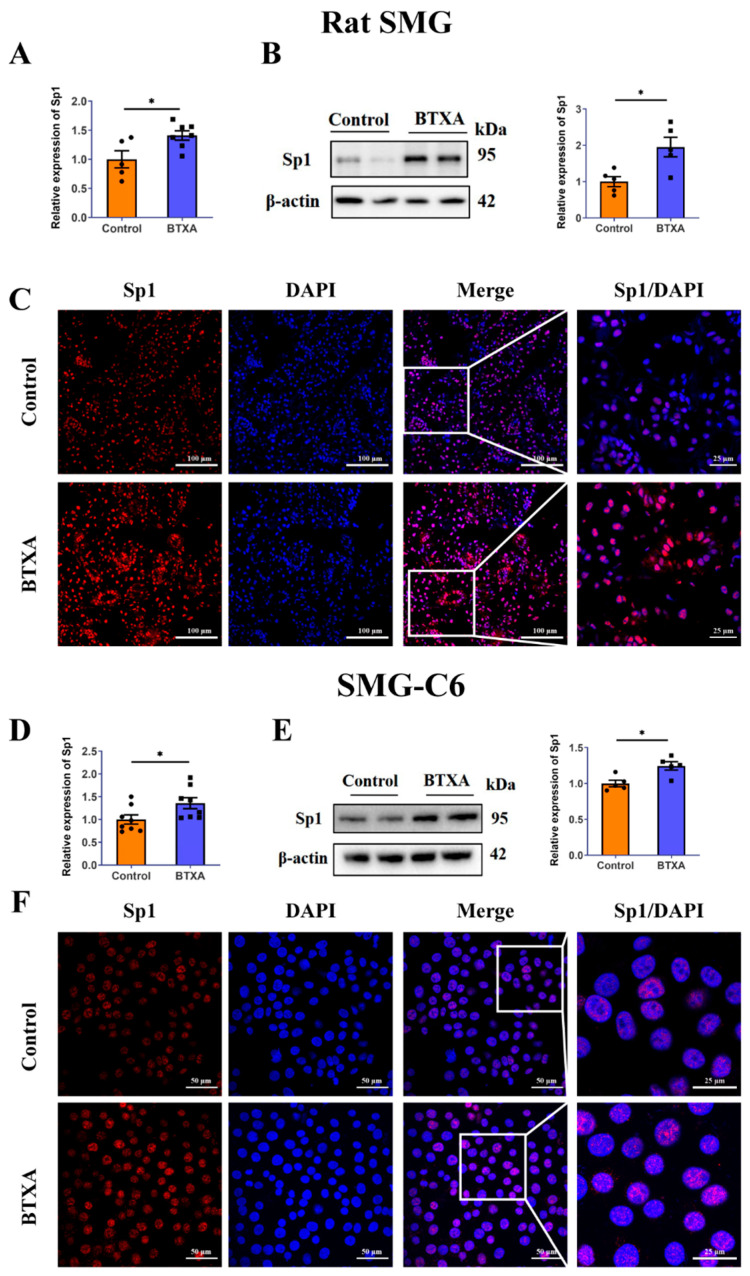
Sp1 is increased in the BTXA-treated submandibular glands and SMG-C6 cells. (**A**) The mRNA levels of Sp1 in rat SMGs. (**B**) The protein levels of Sp1 in rat SMGs. (**C**) Immunofluorescence staining of Sp1 in rat SMGs. (**D**) The mRNA levels of Sp1 in BTXA-treated SMG-C6 cells. (**E**) The protein levels of Sp1 in BTXA-treated SMG-C6 cells. (**F**) Immunofluorescence staining of Sp1 in BTXA-treated SMG-C6 cells. SMG-C6 cells treated with 50 U/mL BTXA for 24 h. SMG, submandibular gland; Sp1, specificity protein-1. Data are presented as mean ± SEM; *n* = 5–8, * *p* < 0.05, compared with control group.

To investigate how the ERK1/2 pathway regulates Cldn1, we examined several Cldn1-related transcription factors (Snail/Slug and Sp1) in BTXA-treated SMG-C6 cells. We found that BTXA increased Sp1 expression (Figure 4E) but did not alter Snail/Slug levels (Figure S1). This finding, coupled with prior studies identifying Sp1 as a downstream target of the ERK1/2 pathway [20,21], led the present study to primarily focus on investigating changes in the transcription factor Sp1. The results of qRT-PCR (Figure 4A) and Western blot (Figure 4B) showed that BTXA increased the expression of Sp1 in rat SMGs. Notably, Sp1 was primarily localized in the nuclei of rat SMGs, and its nuclear staining intensity was significantly increased in the BTXA group (Figure 4C), which was consistent with the Western blot results. Concurrently, we explored the effects of BTXA on the expression and distribution of Sp1 in vitro. The results revealed that BTXA increased the expression of Sp1 in SMG-C6 cells (Figure 4D,E). Immunofluorescence analysis further confirmed that in SMG-C6 cells, Sp1 was predominantly located in the nucleus, and BTXA treatment intensified the nuclear Sp1 staining (Figure 4F).

### 3.5. ERK1/2 Activation Is Involved in the Promotion of Cldn1 Transcription by BTXA Through the Upregulation of Sp1 Expression

**Figure 5:**
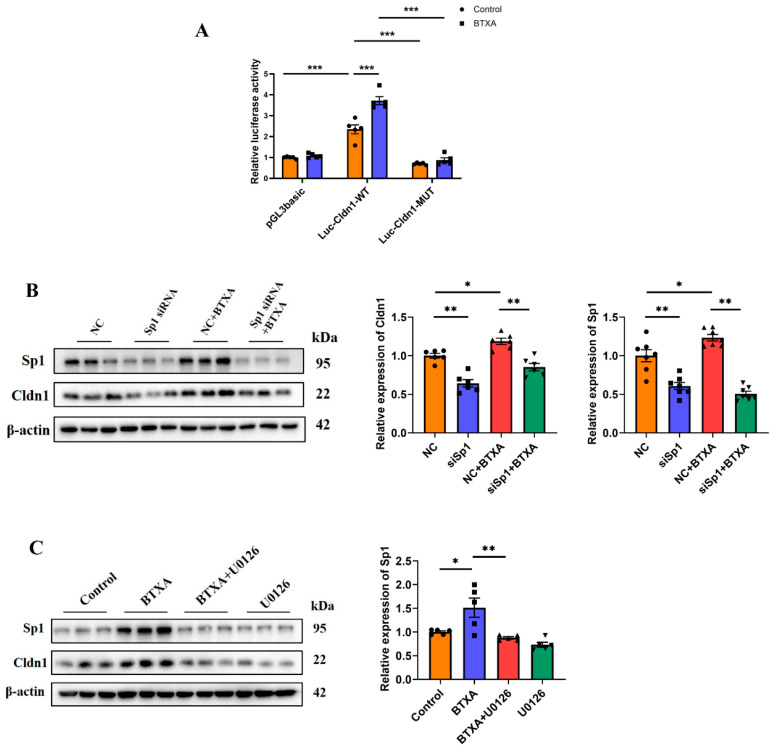
ERK1/2 pathway is activated and involved in BTXA’s promotion of Cldn1 transcription though the upregulation of Sp1 expression. (**A**) Dual-luciferase reporter assay with SMG-C6 cells transfected with a luciferase reporter plasmid. (**B**) Western blot analysis of Sp1 and Cldn1 after transfecting SMG-C6 cells with Sp1-specific siRNA. (**C**) Western blot analysis of Sp1 after preincubating with U0126. SMG-C6 cells treated with 50 U/mL BTXA for 24 h. SP1, specificity protein-1; NC, negative control. Data are presented as mean ± SEM; *n* = 5–7, * *p* < 0.05; ** *p* < 0.01; *** *p* < 0.001 compared with control group.

The results of the ChIP experiment conducted by our research group revealed that, in SMG-C6 cells, Sp1 could bind to the promoter region of Cldn1 at −284 to −84 bp [19]. On this basis, we constructed wild-type binding site plasmids (Luc-Cldn1-WT) and mutant binding site plasmids (Luc-Cldn1-MUT) within the −300 to −75 bp region of the Cldn1 promoter. A luciferase reporter assay revealed that luciferase activity decreased following transfection with Luc-Cldn1-MUT. Moreover, in the Luc-Cldn1-WT group, BTXA significantly increased luciferase activity (Figure 5A), suggesting that BTXA promotes the binding of Sp1 to the promoter region of Cldn1. Furthermore, the use of siRNA to knockdown Sp1 expression helped elucidate the regulatory role of Sp1 in Cldn1. The results showed that the decreased expression of Sp1 effectively reversed the upregulation of Cldn1 induced by BTXA (Figure 5B). These results indicated that Sp1 acted as a transcriptional activator of Cldn1 and that BTXA could increase the transcription of Cldn1 by promoting the binding of Sp1 to the promoter region of Cldn1.

To further verify whether the ERK1/2 pathway regulated the expression of Sp1, we incubated SMG-C6 cells with U0126 and subsequently examined Sp1 expression. The results demonstrated that the upregulation of Sp1 induced by BTXA was effectively reversed (Figure 5C). These findings suggest that ERK1/2 activation is involved in the promotion of Sp1 expression by BTXA, which in turn facilitates Cldn1 transcription.

### 3.6. MiR-124-3p Upregulates Cldn1 Expression by Directly Targeting Sp1 in SMG-C6 Cells

**Figure 6:**
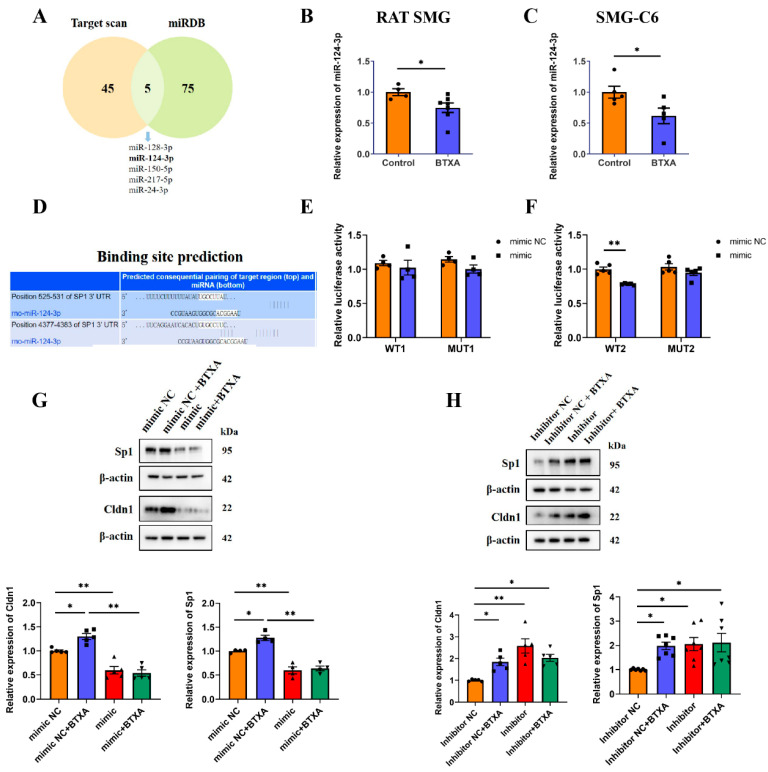
MiR-124-3p upregulates claudin-1 expression by directly targeting Sp1 in SMG-C6 cells. (**A**) Venn diagram showed the intersection of TargetScan and miRdb databases for predicted miRNA regulation of Sp1. (**B**,**C**) The expression levels of miR-124-3p in BTXA-treated rat SMGs and SMG-C6 cells. (**D**) Bioinformatics analysis revealed that the 3′-UTR of Sp1 mRNA contains two conserved binding sites for miR-124-3p. (**E**,**F**) Dual-luciferase reporter assay was performed in SMG-C6 cells co-transfected with a luciferase reporter plasmid and miR-124-3p mimic. (**G**,**H**) Western blot analysis of Sp1 and Cldn1 after transfecting SMG-C6 cells with miR-124-3p mimic (**G**) or inhibitor (**H**). SMG-C6 cells treated with 50 U/mL BTXA for 24 h. Data are presented as mean ± SEM; *n* = 4–7, * *p* < 0.05; ** *p* < 0.01 compared with control group.

MiRNAs serve as a regulatory mechanism for the expression of TJ proteins. By analyzing potential miRNAs that regulate Sp1 via the TargetScan and miRDB databases, we identified an intersection of five miRNAs: miR-128-3p, miR-124-3p, miR-150-5p, miR-217-5p, and miR-24-3p (Figure 6A). High-throughput sequencing revealed that BTXA downregulated the expression of miR-124-3p [17], a finding further validated by qRT-PCR in both rat SMGs and SMG-C6 cells (Figure 6B,C). The TargetScan database showed that the Sp1 3′-UTR contained two conserved binding sites for miR-124-3p (Figure 6D). To investigate these interactions, we constructed wild-type (Luc-Sp1-3′UTR-WT) and mutant (Luc-Sp1-3′UTR-MUT) plasmids. Luciferase reporter assays indicated that the miR-124-3p mimic reduced luciferase activity in cells transfected with the Luc-Sp1-3′-UTR-Wt plasmid, specifically at the second binding site located at nucleotides 4377-4383 of the Sp1 3′UTR (Figure 6E,F). This finding suggests that Sp1 is a direct target of miR-124-3p in SMG-C6 cells. Furthermore, overexpression of miR-124-3p reduced Sp1 and Cldn1 expression, reversing the BTXA-induced upregulation of these proteins (Figure 6G). Conversely, inhibition of miR-124-3p led to increased Sp1 and Cldn1 expression in SMG-C6 cells (Figure 6H).

### 3.7. ERK1/2 Activation Is Involved in BTXA-Induced Downregulation of miR-124-3p

Our previous results revealed that, on the one hand, BTXA could upregulate the expression of Sp1 by activating the ERK1/2 pathway, thereby promoting the transcription of Cldn1. On the other hand, BTXA could promote the transcription of Cldn1 by downregulating the expression of miR-124-3p, leading to the upregulation of its target gene, Sp1. Both the ERK1/2 pathway and miR-124-3p were involved in the upregulation of Sp1 expression by BTXA to promote Cldn1 transcription. However, whether there was a connection between ERK1/2 activation and the downregulation of miR-124-3p expression required further research. Therefore, we conducted the following experiments.

**Figure 7:**
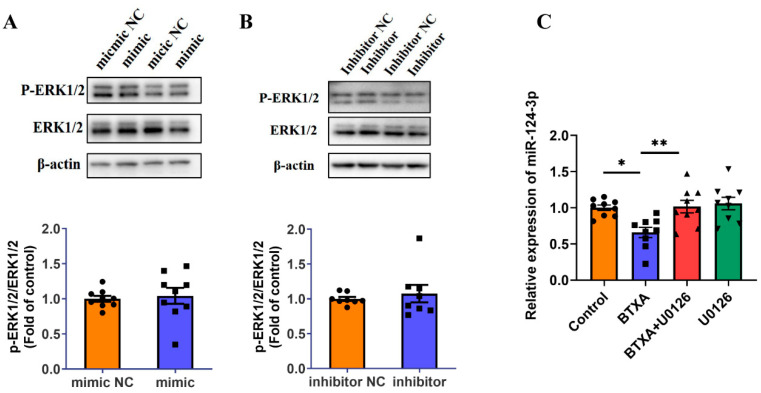
ERK1/2 pathway is activated and involved in BTXA’s downregulation of miR-124-3p expression. (**A**,**B**) Western blot analysis of ERK1/2 phosphorylation after transfecting SMG-C6 cells with miR-124-3p mimic (**A**) or inhibitor (**B**). (**C**) The expression levels of miR-124-3p after preincubating with U0126. SMG-C6 cells treated with 50 U/mL BTXA for 24 h. Data are presented as mean ± SEM; *n* = 8–9, * *p* < 0.05; ** *p* < 0.01 compared with control group.

First, we transfected cells with a miR-124-3p mimic or inhibitor and assessed the phosphorylation status of ERK1/2 via Western blot. The results showed that neither the miR-124-3p mimic nor the inhibitor affected ERK1/2 phosphorylation (Figure 7A,B). Additionally, inhibition of the ERK1/2 pathway using U0126 reversed the BTXA-induced reduction in miR-124-3p expression (Figure 7C). These findings suggest that BTXA may downregulate the miR-124-3p expression by activating the ERK1/2 pathway.

## 4. Discussion

In the present study, we elucidated the crucial role of Cldn1 in BTXA-inhibited SMG secretion. BTXA decreased miR-124-3p expression through the ERK1/2 pathway, which increased the expression of the transcription factor Sp1 and regulated Cldn1 content by facilitating Sp1 binding to the Cldn1 promoter, thereby reducing paracellular permeability. These findings highlight the pivotal role of Cldn1 in the regulation of the TJ barrier by BTXA and reveal a novel mechanism by which BTXA suppresses salivary secretion.

Claudins are crucial transmembrane components of TJs that form both paracellular barriers and pores, playing a key role in determining the permeability of epithelial and endothelial cells [22]. Claudin-1, the first member of the Cldn family, is expressed in a variety of tissues, including the salivary glands, intestine, spleen, brain, kidneys, and testes. Studies have shown that Cldn1 functions primarily as a barrier [23]. Overexpression of Cldn1 in MDCK cells increases the TER value and decreases the paracellular flux [24]. Abnormalities in Cldn1 expression may lead to TJ dysfunction, causing various pathologies. Claudin-1 is essential for the epidermal barrier; knockout mice lacking Cldn1 lose their epidermal granular layer, which serves as a barrier to water and macromolecules, and die of dehydration in the neonatal period [9]. Downregulation of Cldn1 has also been reported in diseases associated with dry skin, such as atopic dermatitis [25] and psoriasis [26]. Silencing of Cldn1 in hepatocytes increases paracellular permeability and has been linked to neonatal ichthyosis–sclerosing cholangitis (NISCH) syndrome [11]. To date, reports on the function of Cldn1 in the salivary glands are limited. Previous studies have shown that Cldn1 functions as a barrier in SMG-C6 cells [19]; our study confirmed these findings, demonstrating that the knockdown of Cldn1 in SMG-C6 cells led to decreased TER and increased FITC-dextran flux. In addition, Cldn1 knockdown reversed the BTXA-induced increase in TER and decrease in FITC-dextran flux, further elucidating the importance of Cldn1 in BTXA-induced inhibition of paracellular permeability in SMG-C6 cells.

MiRNAs have recently been reported to directly or indirectly regulate TJ protein expression and affect epithelial barrier function [27]. Interleukin-1B disrupts intestinal TJ barrier function by upregulating the expression of miR-200C-3p, which subsequently targets and degrades occludin mRNAs [28]. In irritable bowel syndrome, the expression of miR-125b-5p and miR-16 is downregulated, leading to increased expression of ZO-1 and Cldn2, and consequently impairing intestinal epithelial barrier function [29]. MiR-155-5p can indirectly reduce the expression of Cldn1 and occludin by binding to protein kinase inhibitor α, thereby increasing skin barrier permeability and contributing to the onset of atopic dermatitis [30]. On the basis of the miRNA-seq results, we found that BTXA reduced miR-124-3p expression in SMGs and SMG-C6 cells, suggesting that miR-124-3p might be involved in the process by which BTXA inhibited salivary secretion. Aberrant expression of miR-124-3p has been implicated in various biological functions, including apoptosis, metastasis, drug resistance, neural function recovery, adipogenic differentiation, and wound healing [31]. Furthermore, miR-124-3p has been known to regulate the initiation and progression of several cancers, such as hepatocellular carcinoma, gastric cancer, bladder cancer, ovarian cancer, and leukemia. It has also been extensively studied in numerous neurological diseases, including Parkinson’s disease, dementia, and Alzheimer’s disease [31]. MiR-124-3p is known to influence various cellular processes by targeting different genes. For example, enhancer of zeste homolog 2, a target of miR-124-3p, is involved in cell proliferation and migration [32]. Similarly, early growth response 1 (EGR1) is another target, where miR-124-3p downregulates EGR1 to inhibit apoptosis [33]. These findings suggest that miR-124-3p may have a broader impact on modulating cellular behavior within the salivary gland. However, the specific role of miR-124-3p in the physiological and pathological processes of the salivary glands remains unclear. In our experiments, an miR-124-3p mimic decreased Cldn1 expression and reversed the BTXA-induced upregulation of Cldn1, whereas an miR-124-3p inhibitor increased Cldn1 expression. These results demonstrate that miR-124-3p negatively regulates Cldn1 expression and plays a significant role in enhancing the barrier function of SMG-C6 cells via BTXA.

MiRNAs typically exert their negative regulatory effects by binding to the 3′UTRs of target mRNAs. To investigate how miR-124-3p negatively regulate Cldn1, we performed bioinformatic analyses and predicted that miR-124-3p directly bound to Sp1 rather than Cldn1. Sp1, the first identified transcription factor in the specific protein/Kruppel-like factor family, has been reported to promote Cldn1 expression in the intestinal epithelium [34] and human breast cancer epithelium [35]. Our previous studies have also confirmed that Sp1 binds to the Cldn1 promoter in SMG-C6 cells, as demonstrated by ChIP assays [19]. The present study confirmed that BTXA increased Sp1 expression in both SMGs and SMG-C6 cells, with Sp1 predominantly localized in the nucleus. Earlier ChIP assays by our group identified that the Sp1 binding site on the Cldn1 promoter in SMG-C6 cells is between −284 and −84 bp [19], which aligns with binding sites found in the intestinal epithelium (−133 to −61 bp) [34] and human breast cancer epithelium (−138 to −76 bp) [35]. Our dual-luciferase reporter assays revealed that within this binding domain, BTXA enhanced the binding of Sp1 to the Cldn1 promoter region in SMG-C6 cells. To further elucidate the role of Sp1 in the regulation of Cldn1 by BTXA, we used siRNA to knock down Sp1 expression. The results showed that Sp1 knockdown inhibited Cldn1 expression and reversed the BTXA-induced upregulation of Cldn1. These findings demonstrate that Sp1 functions as a transcriptional activator of Cldn1 in submandibular gland acinar cells and suggest that BTXA promotes Cldn1 transcription by upregulating Sp1 expression.

Database predictions revealed that miR-124-3p could bind to Sp1 at two specific binding sites. Studies have confirmed that in H9C2 cardiomyocytes [36] and rat neural stem cells [37], miR-124-3p targets the second binding site (4377–4383) on Sp1. Our dual-luciferase reporter assays further verified that Sp1 was a target gene of miR-124-3p in SMG-C6 cells, with binding occurring precisely at the second site, as noted in the literature. Furthermore, we transfected SMG-C6 cells with a miR-124-3p mimic or inhibitor to assess its regulatory effect on Sp1 expression. The results showed that the miR-124-3p mimic decreased Sp1 expression and reduced BTXA-induced Sp1 upregulation, whereas the miR-124-3p inhibitor led to an increase in Sp1 expression. These findings further confirmed that Sp1 functioned as a target of miR-124-3p in SMG-C6 cells, and that BTXA downregulated miR-124-3p expression, leading to increased expression of its target gene Sp1, thereby promoting Cldn1 transcription.

Studies have shown that MAPK pathway is essential for regulating TJs in SMG. Capsaicin induces occludin translocation from the membrane to the cytosol through the ERK1/2 pathway in SMG-C6 cells. ERK1/2 is required for AMPK-modulated Cldn4 phosphorylation and redistribution [38]. In addition, the ERK1/2/slug signaling axis is involved in TNF-α-induced Cldn3 downregulation and redistribution [39]. These studies underscore the significant role of ERK1/2 in modulating the TJ barrier in the SMG, suggesting that its regulatory effects may vary with different stimuli. Previous studies found that BTXA regulates the MAPK pathway by upregulating the expression of Rac1, RhoA, and Cdc42 in SD rats, thereby increasing angiogenesis [40]. In our previous transcriptomic study, BTXA may target cell surface receptors (e.g., Pdgfrb), adaptor proteins (e.g., Grb2), and MAP3K kinases (e.g., Map3k7), thereby modulating the downstream MAPK signaling pathway and contributing to BTXA-induced inhibition of salivary secretion. Our study confirmed that BTXA activated the ERK1/2 pathway, and that preincubation with an ERK1/2 inhibitor abolished the BTXA-induced increase in Cldn1 expression and decrease in paracellular permeability. Additionally, studies have demonstrated that Sp1 is a downstream target of the ERK1/2 signaling pathway [20,21]. We further found that inhibition of the ERK1/2 pathway reversed the BTXA-induced increase in Sp1 expression, suggesting that BTXA promoted Cldn1 transcription and reduced paracellular permeability by upregulating Sp1 expression through activation of the ERK1/2 pathway.

MiRNA transcription generates a primary transcript called the pri-miRNA, which is then processed by the ribonucleases Drosha and Dicer and subsequently exported from the nucleus to form the mature miRNA [41]. Additionally, studies have shown that the MAPK pathway can regulate miRNA biogenesis, specifically through the ERK1/2 pathway which may inhibit miRNA expression. For example, ERK1/2 increases the expression of the key effector c-Myc, which in turn inhibits the transcription of pri-miRNAs. ERK1/2 phosphorylates exportin 5 (XPO5), inhibiting the export of pre-miRNA from the nucleus to the cytoplasm and consequently downregulating miRNA expression [42]. Here, we found that preincubation with ERK1/2 kinase inhibitors reversed the BTXA-induced downregulation of miR-124-3p expression, which suggested that BTXA might reduce miR-124-3p expression by activating the ERK1/2 pathway, thereby affecting paracellular permeability. These results indicate that the ERK1/2 pathway is an essential signaling pathway involved in the regulation of the TJ barrier by BTXA.

In the present study, we identified a novel mechanism by which BTXA inhibits salivary secretion by using a combination of animal and cell experiments. However, our study has several limitations. First, our experiments were conducted using rat SMGs instead of human SMGs. Human SMGs are composed of a mixture of serous and mucous acini, whereas rat glands consist of seromucous acini. Second, we did not investigate the mechanism underlying the BTXA-induced upregulation of Cldn3. Future research should focus on this topic to provide a more comprehensive understanding of the mechanisms underlying the BTXA-induced suppression of salivary secretion.

## 5. Conclusions

Our study demonstrated that Cldn1 is a crucial target through which BTXA enhances the barrier function of SMG acinar epithelial cells. We revealed a novel mechanism by which BTXA downregulates miR-124-3p expression via activation of the ERK1/2 pathway, subsequently targeting Sp1 to promote Cldn1 transcription and strengthen the epithelial barrier. These findings expand our understanding of miRNA functions in salivary secretion and provide a theoretical foundation for the clinical application of BTXA.

## Figures and Tables

**Table 1:** Sequences of claudin-1 and Sp1 siRNA.

Gene	Sequence (5′-3′)
rno-claudin-1-siRNA	Forward GCCACAGCAUGGUAUGGAATT
	Reverse UUCCAUACCAUGCUGUGGCTT
rno-Sp1-siRNA	Forward GCAAGUUCUGACAGGUCUATT
	Reverse UAGACCUGUCAGAACUUGCTT
negative control (NC)	Forward UUCUCCGAACGUGUCACGUTT
	Reverse ACGUGACACGUUCGGAGAATT

**Table 2:** Primer sequences used in real-time PCR.

Gene	Forward Primer (5′-3′)	Reverse Primer (5′-3′)
Sp1	GGACAGTTGAGCAGCATT	CCATCATCATTCGGACAC
β-actin	GAGACCTTCAACACCCCAGCC	TCGGGGCATCGGAACCGCTCA
miR-124-3p	GCGTAAGGCACGCGGTG	AGTGCAGGGTCCGAGGTATT
U6	CTCGCTTCGGCAGCACA	AACGCTTCACGAATTTGCGT

## Data Availability

The data presented in this study are available on request from the corresponding authors.

## Supplementary Materials

The following supporting information can be downloaded at: https://www.mdpi.com/article/10.3390/cells14171366/s1, Figure S1: Effect of BTXA on Snail and Slug expression in SMG-C6 cells.
