# Supplementary figures and images for: Botulinum Toxin Type A Inhibits Submandibular Secretion via the ERK/miR-124-3p/Specificity Protein 1/Claudin-1 Axis
Source: Cells. 2025 Sep 2;14(17):1366. doi: 10.3390/cells14171366 (PMC12428761; doi:10.3390/cells14171366)

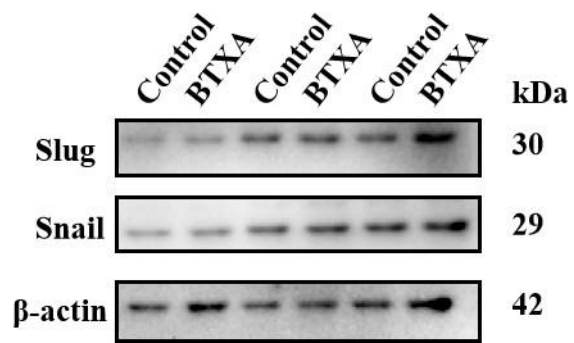

**Figure S1.** Effect of BTXA on Snail and Slug expression in SMG-C6 cells.

Supplement: Supplementary file 1 [file cells-14-01366-s001.zip › cells-3738104-supplementary.pdf]
